# Central features in health-related quality of life in older adults: network analysis using nationwide survey data

**DOI:** 10.1192/bjo.2023.536

**Published:** 2023-08-08

**Authors:** Eun Jung Cha, Yeonsil Moon, Seung-Ho Ryu, Hong Jun Jeon

**Affiliations:** Department of Psychiatry, Konkuk University Medical Center, Konkuk University School of Medicine, Seoul, Republic of Korea; Department of Neurology, Konkuk University Medical Center, Konkuk University School of Medicine, Seoul, Republic of Korea

**Keywords:** Quality of life, ageing, health, exercise, stress

## Abstract

**Background:**

Population ageing is a global phenomenon that necessitates consideration of health-related quality of life (HRQoL) in older adults. Previous studies have investigated related factors including mobility, social support and living situations.

**Aims:**

This study aimed to provide a network perspective on factors related to HRQoL.

**Method:**

Cross-sectional nationwide data were obtained from the Korean National Health and Nutrition Examination Survey conducted from 2018 to 2020 for network analyses. Data for participants aged 65 years or above were analysed, resulting in a total of 4317 eligible cases. The variables included were EQ-5D (a measure of HRQoL), household income, education, living situation, subjective perceived health, Charlson Comorbidity Index (a measure of medical comorbidities), stress, exercise per week, alcohol consumption and smoking. Three networks were produced: (a) EQ-5D dimensions network, (2) EQ-5D dimensions, lifestyle and psychosocial factors network, and (3) overall EQ-5D index, lifestyle and psychosocial factors network. Node centralities, bridge centralities and edges of the networks were examined.

**Results:**

The most central EQ-5D dimension was the ability to carry out usual activities. In the second network, subjective health, stress and anxiety/depression were revealed as nodes with high bridge centralities. Subjective health, exercise, and Charlson Comorbidity Index were nodes closely linked to the overall EQ-5D index.

**Conclusions:**

The results emphasise the importance of enhancing functional independence and subjective health cognition, increasing routine exercise and reducing stress as targets for interventions to improve HRQoL in older adults.

Population ageing has become a global phenomenon, especially in developed countries. In 1990, it was documented that 6% of the world population was aged 65 years or older. By 2019, this statistic had increased to 9%, and it is estimated to further increase to 16% by 2050 worldwide.^1^ This poses many concerns, as older adults tend to be more isolated, perform fewer occupational activities (professional, leisure, etc.), and have limited social support and access to health-related information. Higher exposure to numerous health risks through ageing results in a high socioeconomic burden due to increased medical costs.

The various health risks and their consequences for social welfare in the older adult population can be investigated using a concept termed health-related quality of life (HRQoL). HRQoL was developed to specifically examine health-related aspects of quality of life, such as disease status, occurrence of pain or discomfort, and physical functioning.^2^ Previous studies investigating factors that affect HRQoL in older adults have found physical functions including mobility, grip strength and balance to be significantly associated with HRQoL.^3^ In addition, lower levels of social support predict lower levels of HRQoL, especially for older adults living alone.^4^ These findings suggest that HRQoL is affected by a wide range of variables.

Recently, a psychometric approach called network analysis has been proposed as a method to investigate variable interactions. In comparison with other methods of analysis, this approach offers a broader perspective on variable interactions by producing a visual and easily comprehensible network structure and novel indices to measure patterns of interaction.^5^ In public health, network analysis can be useful in investigating social and environmental influences on health, or health-related factors such as HRQoL. Constructing such networks enables the development of more efficient intervention plans by offering insights regarding target factors for enhancing HRQoL.^6^ To date, no study has investigated HRQoL and related factors in older adults from a network perspective.

Therefore, the major aim of this study was to use network analysis to provide insight into HRQoL and related factors using nationwide health data. First, we constructed a network consisting only of different dimensions of HRQoL to examine which particular dimensions have important roles. Second, we investigated bridging variables that link HRQoL dimensions with various lifestyle and psychosocial factors. Finally, we analysed the relationships connecting the overall HRQoL index with other lifestyle factors to identify variables of primary importance for enhancing HRQoL.

## Methods

### Study design and participants

Data were obtained from the publicly available Korean National Health and Nutrition Examination Survey (KNHANES) for 2018, 2019 and 2020. The KNHANES is conducted by the Korea Disease Control and Prevention Agency in the form of a rolling sample survey.^7^ Rolling sampling is a useful sampling method for large population surveys, in which non-overlapping F number of rolling samples are periodically collected. The probability distribution of each sample is 1/*F*, such that after *F* of cycles, the cumulative samples become a sample of the entire population.

**Fig. 1:**
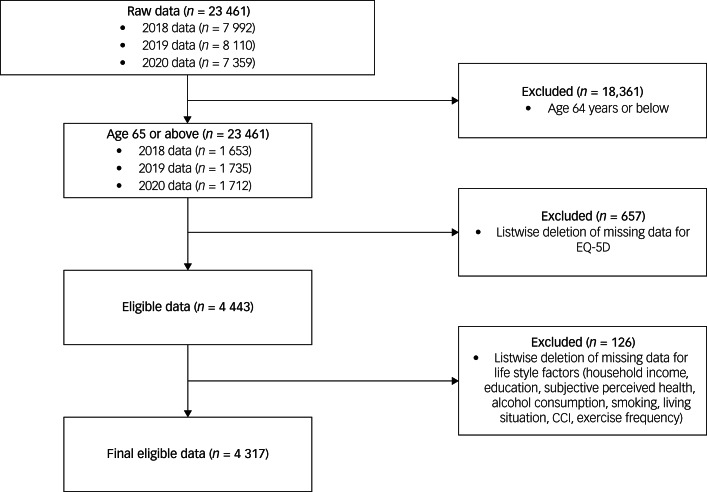
Flow chart illustrating case exclusion process for the 2018, 2019 and 2020 Korean National Health and Nutrition Examination Survey. CCI, Charlson Comorbidity Index.

The 2018 data were taken from the seventh KNHANES, third year (KNHANES VII-3; Korea Disease Control and Prevention Agency),^8^ 2019 data from the eighth KNHANES, first year (KNHANES VIII–1)^9^ and 2020 data from the eighth KNHANES, second year (KNHANES VIII–2).^10^ Selecting cases with ages 65 years or above resulted in 1653, 1735 and 1712 cases (5100 in total) for KNHANES VII-3, VIII-1 and VIII-2, respectively. Listwise deletion excluded 657 cases owing to missing responses. As a result, the final data-set eligible for analysis contained 4317 cases, comprising 1878 males (43.5%) and 2439 females (56.5%). Participants’ mean age was 72.74 (s.d. = 5.07). Figure 1 shows a flow chart depicting the case exclusion process for this study.

The authors assert that all procedures contributing to this work comply with the ethical standards of the relevant national and institutional committees on human experimentation and with the Helsinki Declaration of 1975, as revised in 2008. Consent was received by the Korea Disease Control and Prevention Agency from all participants prior to conducting the KNHANES. All procedures involving human subjects were approved by the Institutional Review Board of Konkuk University Medical Center (KUMC 2022-07-063).

### Measures

#### EQ-5D

The EQ-5D is a self-report scale developed by the EuroQol Group.^11^ The EQ-5D rates HRQoL based on five dimensions: mobility, self-care, usual activities, pain/discomfort and anxiety/depression. Each dimension is rated on a three-point Likert scale ranging from no problem,^1^ some or moderate problem,^2^ and extreme problem.^3,12^ The EQ-5D scale has been translated into Korean and validated for use in that language.^13^ Here, EQ-5D scores were converted into a single index score computed using weighted scores for each dimension, with the weight assignment developed by Lee et al.^14^ The EQ-5D has shown acceptable reliability and construct validity in older adult populations.^15,16^ Permission for the use of the EQ-5D scale was obtained for the KNHANES and can be found in the publicly available data instruction file (https://knhanes.kdca.go.kr/knhanes/sub03/sub03_02_05.do).

#### Lifestyle and psychosocial factors

Household income was assigned a score ranging from 1 (first quartile, low income) to 4 (fourth quartile, high income). Household income was selected instead of individual income, as older adults above 65 are likely to have retired and receive income from sources other than individual work. Education was assigned a score such that higher scores indicated higher educational background, ranging from 1 (primary school or lower) to 4 (university or higher). Living situation was re-coded into a binary variable where 1 indicated living with someone, and 0 indicated living alone. Subjective health asked how participants felt about their health on average, with scores ranging from 1 (very good) to 5 (very bad). The stress item asked how much the subject experienced stress on average. Stress was re-coded such that higher scores indicated a worse condition. As a result, stress scores ranged from 1 (almost none) to 4 (very much). Alcohol consumption frequency scores ranged from 1 (almost none) to 6 (more than four times a week). Smoking was re-coded into a binary variable where 1 indicated a smoker and 0 a non-smoker. Exercise frequency was re-coded into three levels such that 0 indicated no exercise per week, 1 indicated fewer than 3 days per week, and 2 indicated more than 4 days per week. Medical comorbidity was evaluated using the modified version of the Charlson Comorbidity Index (CCI) for this study.^17^ A score of 1 was assigned if the subject responded positively to questions asking whether they were currently suffering from myocardial infarction, angina, stroke, lung disease (tuberculosis, asthma, lung cancer), connective tissue disease (rheumatoid arthritis, osteoarthritis), liver disease (hepatitis B, hepatitis C), diabetes or and kidney disease; a score of 2 was assigned for those with cancer, and 3 for those with liver cirrhosis. CCI index was coded into four levels, ranging from 0 (no comorbidities) to 3 (three or more comorbidities).

### Statistical analysis

#### Descriptive analysis

Prior to the main analyses, we conducted a descriptive analysis using SPSS. For each variable, we calculated frequencies and percentages by gender, age, household income, education, living situation, subjective health, EQ-5D, CCI, stress, alcohol consumption, smoking and exercise. For the EQ-5D index, we computed the mean and standard deviation. The results are presented in Table 1.

**Table 1:** Descriptive statistics of demographic, lifestyle and psychosocial factors among the participants

Variable	Value
Gender	
Male	1878 (43.5%)
Female	2439 (56.5%)
Age, years	72.7 (s.d. = 5.1)
Range	15 (65 to 80)
Median	72
Household income	
First quartile (low)	1944 (45.0%)
Second quartile (medium low)	1228 (28.4%)
Third quartile (medium high)	710 (16.4%)
Fourth quartile (high)	435 (10.1%)
Education	
Primary school or below	2332 (54.0%)
Middle school	725 (16.8%)
High school	819 (19.0%)
University or above	441 (10.2%)
Living situation	
Alone	1019 (23.6%)
With someone	3298 (76.4%)
Subjective health	
Very good	188 (4.4%)
Good	756 (17.5%)
Average	2126 (49.2%)
Bad	865 (20.0%)
Very bad	382 (8.8%)
EQ-5D	
EQL1: Mobility	
No problems in walking about	2769 (64.1%)
Some problems in walking about	1488 (34.5%)
Confined to bed	60 (1.4%)
EQL2: Self-care	
No problems with self-care	3883 (89.9%)
Some problems with self-care	402 (9.3%)
Unable to wash or dress myself	32 (0.7%)
EQL3: Usual activities	
No problems with performing usual activities	3549 (82.2%)
Some problems with performing usual activities	708 (16.4%)
Unable to perform usual activities	60 (1.4%)
EQL4: Pain/discomfort	
No pain or discomfort	2827 (65.5%)
Moderate pain or discomfort	1275 (29.5%)
Extreme pain or discomfort	215 (5.0%)
EQL5: Anxiety/depression	
Not anxious or depressed	3726 (86.3%)
Moderately anxious or depressed	546 (12.6%)
Extremely anxious or depressed	45 (1.0%)
EQ-5D index	0.9 (s.d. = 0.2)
CCI	
0	1955 (45.3%)
1	1548 (35.9%)
2	600 (13.9%)
3 or above	214 (5.0%)
Stress experienced on average	
Almost none	1279 (29.6%)
A little bit	2292 (53.1%)
A lot	591 (13.7%)
Very much	155 (3.6%)
Alcohol consumption frequency during the past year	
Never	2173 (50.3%)
Once or none per month	670 (15.5%)
Once per month	282 (6.5%)
2–4 times per month	523 (12.1%)
2–3 times per week	379 (8.8%)
4 times or more per week	289 (6.7%)
Smoking	
Smoker	3249 (75.3%)
Non-smoker	1068 (24.7%)
Exercise (walking) days per week	
None	1069 (24.8%)
Less than 3 days	1060 (24.6%)
4 days or above	2188 (50.7%)

CCI, Charlson Comorbidity Index.

In addition, we calculated frequencies by gender, age, household income, education, living situation, subjective health and CCI for each level of all EQ-5D dimensions and compared them using chi-squared tests. The results are presented in Table 2.

**Table 2:** Frequencies and chi-squared test results for EQ-5D dimensions according to gender, age, household income, education, living situation, subjective health and CCI

Variable	EQL1: Mobility				EQL2: Self-care				EQL3: Usual activities				EQL4: Pain/discomfort				EQL5: Anxiety/depression			
	1	2	3	χ^2^	1	2	3	χ^2^	1	2	3	χ^2^	1	2	3	χ^2^	1	2	3	χ^2^
Gender																				
Male	1352	509	17	89.96 (*P* < .001)	1732	135	11	19.10 (*P* < .001)	1611	241	26	30.96 (*P* < .001)	1393	444	41	129.62 (*P* < .001)	1691	180	7	44.32 (*P* < .001)
Female	1417	979	43		2151	267	21		1938	467	34		1434	831	174		2035	366	38	
Age, years																				
65–72	1620	563	21	171.74 (*P* < .001)	2070	127	7	79.74 (*P* < .001)	1944	245	15	112.64 (*P* < .001)	1509	617	78	28.51 (*P* < .001)	1934	256	14	12.04 (*p* = .002)
73 or above	1149	925	39		1813	275	25		1605	463	45		1318	658	137		1792	290	31	
Household income																				
First quartile (low)	1043	859	42	193.90 (*P* < .001)	1688	239	17	41.24 (*P* < .001)	1481	423	40	92.15 (*P* < .001)	1157	650	137	73.45 (*P* < .001)	1609	302	33	48.01 (*P* < .001)
Second quartile (medium low)	841	378	9		1131	90	7		1059	160	9		844	345	39		1083	141	4	
Third quartile (medium high)	544	161	5		660	47	3		619	84	7		502	181	27		636	68	6	
Fourth quartile (high)	341	90	4		404	26	5		390	41	4		324	99	12		398	35	2	
Education																				
Primary school or below	1276	1009	47	224.48 (*P* < .001)	2016	294	22	74.96 (*P* < .001)	1802	486	44	99.07 (*P* < .001)	1392	771	169	115.04 (*P* < .001)	1959	334	39	44.81 (*P* < .001)
Middle school	498	219	8		667	54	4		610	107	8		487	214	24		624	97	4	
High school	630	184	5		778	36	5		733	78	8		605	198	16		734	84	1	
University or above	365	76	0		422	18	1		404	37	0		343	92	6		409	31	1	
Living situation																				
Alone	558	432	29	62.96 (*P* < .001)	887	124	8	12.97 (*P* = .002)	775	226	18	34.66 (*P* < .001)	594	350	75	36.17 (*P* < .001)	834	161	24	35.62 (*P* < .001)
With someone	2211	1056	31		2996	278	24		2774	482	42		2233	925	140		2892	385	21	
Subjective health																				
Very good	163	25	0	801.58 (*P* < .001)	181	7	0	456.14 (*P* < .001)	179	8	1	755.13 (*P* < .001)	160	25	3	825.84 (*P* < .001)	175	11	2	424.70 (*P* < .001)
Good	641	114	1		733	21	2		722	33	1		638	117	1		721	35	0	
Average	1511	607	8		2017	102	7		1912	204	10		1556	530	40		1942	178	6	
Bad	362	487	16		706	150	9		563	281	21		368	414	83		655	194	16	
Very bad	92	255	35		246	122	14		173	182	27		105	189	88		233	128	21	
CCI																				
0	1490	453	12	257.85 (*P* < .001)	1841	107	7	107.32 (*P* < .001)	1745	197	13	165.84 (*P* < .001)	1488	417	50	217.09 (*P* < .001)	1779	162	14	72.00 (*P* < .001)
1	895	625	28		1376	160	12		1235	292	21		935	523	90		1294	236	18	
2	292	293	15		493	95	12		424	155	21		300	246	54		482	109	9	
3 or above	92	117	5		173	40	1		145	64	5		104	89	21		171	39	4	

CCI, Charlson Comorbidity Index.

#### Network I: EQ-5D network

To investigate how each dimension of EQ-5D contributed to the overall network, we first conducted network analysis on the five dimensions of EQ-5D to investigate its structure; this was termed ‘Network I’. In a network, variables are each represented as nodes (circles) and the relationships between the nodes as edges (lines). As our data were ordinal, we created a polychoric correlation matrix to compute the network. Edges were computed using partial correlation coefficients between nodes. Centralities are indices characteristic to networks, each representing a pattern of node interaction. Strength, closeness and betweenness centrality indices for each node were computed. Strength is a measure of how strongly a node is related to each adjacent node, determined by considering the absolute values of edge weights. Closeness refers to the inverse sum length of the shortest path of a node to all other nodes in the network. Finally, betweenness is the number of times a node of interest is passed through on the shortest route between every possible pair of nodes in the network.^18,19^ All analyses involving network analyses were conducted in R studio (version 4.2.0.), a development environment for the programming language R. Network analyses were conducted by regularised estimation using graphical least absolute shrinkage and selection operator with the extended Bayesian information criteria.^20,21^ All network and centrality analyses were conducted using the qgraph package.^22^ Detailed methods and explanations of network analysis are provided in Supplementary Text 1 available at https://doi.org/10.1192/bjo.2023.536.

#### Network II: bridge analysis between EQ-5D and lifestyle and psychosocial factors

Our second aim was to identify the bridging nodes connecting EQ-5D dimensions and lifestyle factors. Nodes were grouped into two communities: EQ-5D dimensions, comprising five nodes; and lifestyle and psychosocial factors, comprising nine nodes. The resulting network was termed ‘Network II’. To identify bridging nodes, we computed bridge centrality indices. Bridge centralities are defined similarly to extant centrality indices but in the context of node communities. For example, in case of a network with two communities of nodes, a node having high bridge strength indicates that it has the largest sum absolute value of edges connecting to nodes in the other community.^23^ However, for our network, we computed bridge expected influence instead of bridge strength, as this also takes negative edges into account.^24^ Bridge centrality analyses were conducted using R, with package networktools.^25^ Details regarding how bridge centrality was computed are available in Supplementary Text 1.

#### Network III: edge analysis between EQ-5D index and lifestyle and psychosocial factors

The third network, ‘Network III’, was constructed to examine which lifestyle and psychosocial factors were strongly related to the overall HRQoL level. As such, the five nodes representing EQ-5D dimensions were replaced with a single node representing the EQ-5D index. To investigate edges connected to the EQ-5D index, we used the bootnet function to conduct significance testing on edges connecting the EQ-5D node to other nodes.^26^

#### Accuracy and stability analysis for all estimations

We performed bootstrapping to evaluate the accuracy and stability of all parameter estimations. Edge weights of the network were evaluated by estimating the 95% confidence interval for each edge using a non-parametric bootstrap method with 1000 bootstraps. Next, the reliability of centralities was evaluated by computing correlation stability coefficients (CS-coefficients) for each centrality. Detailed explanations of CS-coefficients are provided in Supplementary Text 1. The recommended cut-off for CS-coefficients is 0.5, and it is advised not to interpret centralities scoring CS-coefficients below 0.25.^26^

To evaluate the edge weights, a bootstrapped difference test was performed. This test involves a null-hypothesis significance test to compare whether one edge weight significantly differs from another, based on their bootstrapped CI.^26^

## Results

### Descriptive statistics

Around half of the participants reported low household income (45.0%) and indicated their highest level of education to be primary school or below (54.0%). The majority of the participants were living with someone (76.4%). Most participants indicated their health condition to be average (49.2%). On the CCI index, 45.3% scored 0, indicating no comorbidities, whereas 35.9% scored 1, 13.9% scored 2, and 5% scored 3 or above. In addition, 50.3% of participants indicated that they did not consume any alcohol during the past year, 75.3% were currently smokers and 50.7% reported that they exercised 4 days or more per week. A significant proportion of participants reported experiencing almost no stress (29.6%) or some level of stress (53.1%) on average.

The majority of participants indicated that they had no problems in performing self-care (89.9%) and usual activities (82.2%) and did not feel anxious or depressed (86.3%). A substantial proportion of participants indicated that they were experiencing some problems with walking about (34.5%) and moderate pain or discomfort (29.5%). The overall mean for the EQ-5D index was 0.89 (s.d. = 0.15). A summary of descriptive statistics for EQ-5D and lifestyle factors is presented in Table 1.

### Network I

**Fig. 2:**
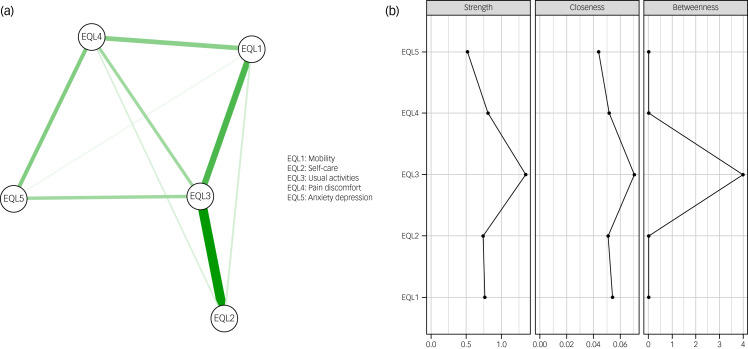
(a) Network I containing EQ-5D dimensions. Thicker lines indicate stronger edge weights. All edges represent positive partial correlation coefficients. (b) Graph showing raw centrality scores for strength, closeness and betweenness for EQ-5D dimensions.

Network I is visualised in Fig. 2a. The strongest edge was that connecting EQL2 (self-care) and EQL3 (usual activities), with partial correlation coefficient value 0.55. The bootstrapped CI results for each edge are presented in Supplementary Fig. 1. Details of network visualisation are given in Supplementary Text 1.

According to the centrality analysis, overall, the largest centralities were observed for EQL3 (usual activities). Figure 2b illustrates the raw centrality scores for strength, closeness and betweenness. As all CS-coefficients were above 0.5, all indices were treated as accurate and were thus interpreted. The case-dropping bootstrap results for the centrality indices can be found in Supplementary Fig. 2.

### Network II

**Fig. 3:**
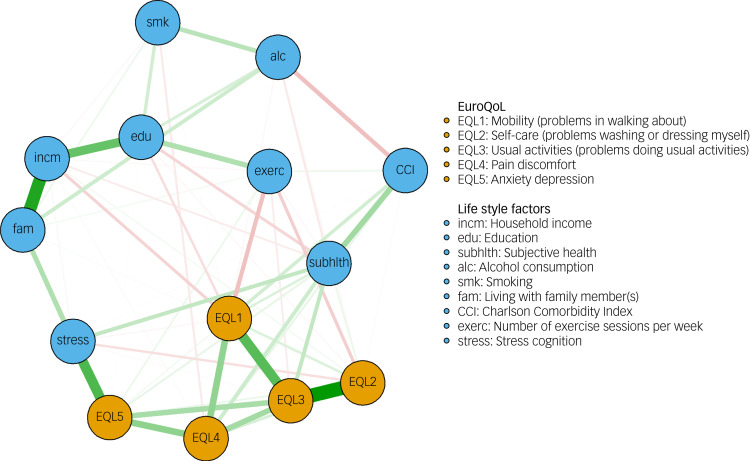
Network II showing EQ-5D dimensions and lifestyle factors. Thicker lines indicate stronger edge weights. Red indicates negative edge weights, and green indicates positive edge weights. EQ-5D dimension nodes are shown in orange, and lifestyle factor nodes in blue.

Network II contained all five dimensions of EQ-5D as well as lifestyle and psychosocial factors and was used to examine bridging nodes. Network II is illustrated in Fig. 3. Of the 91 possible total edges that could be estimated, 63 edges remained in the network. The strongest edge weight connected EQL2 and EQL3, with a partial correlation coefficient value of 0.52. Some negative edges were observed (e.g., EQL1–exercise). Bootstrapped CIs for all edges are presented in Supplementary Fig. 3.

In the bridge centrality analysis, stress had the highest bridge closeness value, followed by subjective health. Stress also had the highest value of bridge betweenness, followed by EQL5 (anxiety/depression), and family (living situation). Subjective health had the highest bridge two-step expected influence, followed by EQL5 and stress. A graph illustrating raw scores of bridge centralities is shown in Supplementary Fig. 4. CS-coefficients for bridge centralities were all above 0.5. Thus, all bridge centralities were interpreted. Results of the case-dropping bootstrap can be found in Supplementary Fig. 5.

### Edge analysis

**Fig. 4:**
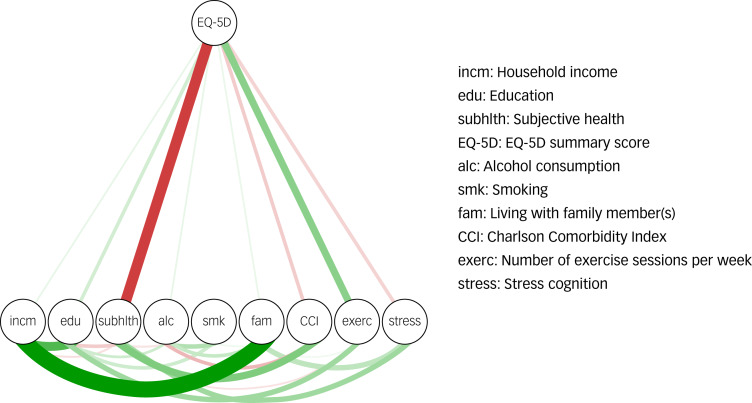
Network III constructed using the flow function from the qgraph package. Edge weights were derived from the EQ-5D index. Thicker lines indicate stronger edge weights. Red indicates negative edge weights, green indicates positive edge weights.

Finally, for Network III, edges connected to the EQ-5D index were investigated. The resulting network is shown in Supplementary Fig. 6. As our main interest was the EQ-5D node, we used the flow function included in the qgraph package^22^ to create a diagram showing edges stemming from the EQ-5D index (Fig. 4). Bootstrapped edge-weight CIs are shown in Supplementary Fig. 7. The strongest edge connected with the EQ-5D index was for subjective health, followed by exercise.

## Discussion

This study investigated Korean nationwide health data for an older adult population with three aims. First, we investigated which node had the central role in a network of HRQoL dimensions. The results revealed EQL3 (usual activities) to have the highest centrality index within the HRQoL network. Second, bridge analysis showed that subjective health, stress and EQL5 (anxiety/depression) had the highest bridge centrality indices. Finally, in Network III, subjective health and exercise were found to be strongly related to the EQ-5D index. Our results have implications regarding HRQoL in the older adult population, which we discuss below.

Usual activities (EQL3) emerged as the most central node, with the highest strength, closeness and betweenness centralities. This suggests that EQL3 has the strongest and closest links with other EQ-5D dimensions. Within the EQ-5D framework, EQL3 is an important index designed to capture activities involved in daily life, such as work, housework, family and leisure activities.^27^ It often emerges as an important dimension in studies that investigate HRQoL in patients, for example, those with chronic conditions.^28,29^ EQL3 can also be referred to as functional independence, defined as one's ability to perform activities of daily living. Functional independence ensures autonomy and is thus an important indicator of older adults’ quality of life.^30,31^ In cases of degenerative diseases that are common in older adults, such as dementia, interventions are often planned to tackle the decline in functional independence, as this is frequently linked to other problematic conditions such as depression, falls and cardiovascular diseases.^32^ Furthermore, this result is in line with a previous study that showed functional independence to be a determinant of HRQoL dimensions, including usual activities.^33^ This result highlights the importance of functional independence for older adults and supports the use of various types of aid to maintain functional independence. For example, information technology, mobile phone applications and the Internet of Things are being developed^34,35^ to improve functional independence in older adults. In this regard, improvements in smart home technologies that can provide assistance, safety and convenience should be emphasised as a step towards enhancing functional independence in older adults. The results of our study suggest that improving functional independence is a promising direction towards subsequently improving other dimensions of HRQoL in the older adult population.

Another important result of this study was the high bridge closeness and bridge expected influence of subjective health in Network II. Bridge closeness is a measure of the average distance from a node to all nodes in another community, and bridge expected influence is defined as the sum of edges a node has with nodes from the other community. Thus, subjective health may have a strong and immediate effect on all dimensions of EQ-5D. This was emphasised in Network III, where EQ-5D index and subjective health showed a strong negative association. It should be noted that subjective health is a subjective opinion of what an individual thinks of their health on average, whereas each dimension of EQ-5D is descriptive and thus relatively objective. Previous studies have shown that perceived health is significantly worse in older adults compared with younger adults,^36^ especially in those with lower levels of income, education and social support, and increased depression.^37–39^ Low subjective health in older adults is known to be a predictor of mortality and functional decline.^40,41^ Therefore, to improve subjective health in medical services targeted to older adults, these predictive and associated factors should be considered. Our results indicate that improvements in subjective health will have positive effects on HRQoL.

Our results also showed stress and regular exercise to be important bridge factors connecting lifestyle factors and HRQoL. Stress disrupts homeostasis, leading to negative effects on one's health.^42^ Therefore, it is not surprising that among many lifestyle and psychosocial factors, stress had a close association with HRQoL. The result for exercise indicates that the importance of exercise in older adults cannot be underestimated. Interventions for physical training in older adults have been shown to have positive effects on cognitive functions, mood and dementia.^43,44^ Furthermore, one previous study has shown psychological distress to be a significant mediator between moderate-to-vigorous physical activity and quality of life.^45^ Combined with our results, this specifies the direction of change that cannot be observed in a network. These results highlight the role of exercise as a variable that initiates positive changes in HRQoL and stress. Exercise improves functional independence by preventing ageing and increasing subjective health perception and is also a good means of reducing stress.^46–48^ However, the accessibility of exercise to older adults is limited, as they may require specialised methods of exercise because of physical limitations due to ageing. Therefore, the development of specialised programmes in institutions such as senior citizen centres is necessary to promote regular exercise in the older adult population and enhance HRQoL.

This study had a number of limitations. First, data analysis was conducted under a cross-sectional design. Therefore, it was difficult to establish directionality between variables, although for some variables, the causal direction may have been self-explanatory (e.g. living situation and household income). Second, although one of our main results focused on subjective health cognition, only a single item measured this concept. This limitation highlights the need for development of tools to evaluate subjective health cognition in further detail. Third, other important psychosocial and lifestyle factors such as nutrition status, social capital and marital status were not included, despite their possible effects on HRQoL. Finally, although there are other factors that are known to affect HRQoL in older adults, such as cognitive dysfunction^49^ and social isolation,^50^ only a limited number of factors were taken into account owing to the retrospective nature of this study.

Despite these limitations, we obtained meaningful results by evaluating a network comprising factors that have been known to affect HRQoL in older adults using community-based, large-scale nationwide data. To improve HRQoL in older adults, functional independence should be considered a priority target in health policies. Efforts should be made to enhance subjective health cognition via education and psychological interventions rather than considering it to be an individual characteristic. Our results suggest that intervening with respect to modifiable factors such as stress, subjective health and regular exercise may be sufficient enough to increase HRQoL.

## Supporting information

Cha et al. supplementary material Cha et al. supplementary material

## Data Availability

The data-sets used during the current study are available from the homepage of the Korea Disease Control and Prevention (https://knhanes.kdca.go.kr/knhanes/sub03/sub03_01.do). They can also be made available upon reasonable request to the corresponding author, H.J.J. The code used for analysis in this study is available for public access (https://cran.r-project.org/web/packages/qgraph/index.html).
