# Supplementary material for: Central features in health-related quality of life in older adults: network analysis using nationwide survey data
Source: BJPsych Open. 2023 Aug 8;9(5):e145. doi: 10.1192/bjo.2023.536 (PMC10617500; doi:10.1192/bjo.2023.536)
Supplement: Cha et al. supplementary material [file S2056472423005367sup001.pdf]

Figure S1

Bootstrapped 95% confidence intervals for all edges in Network I.

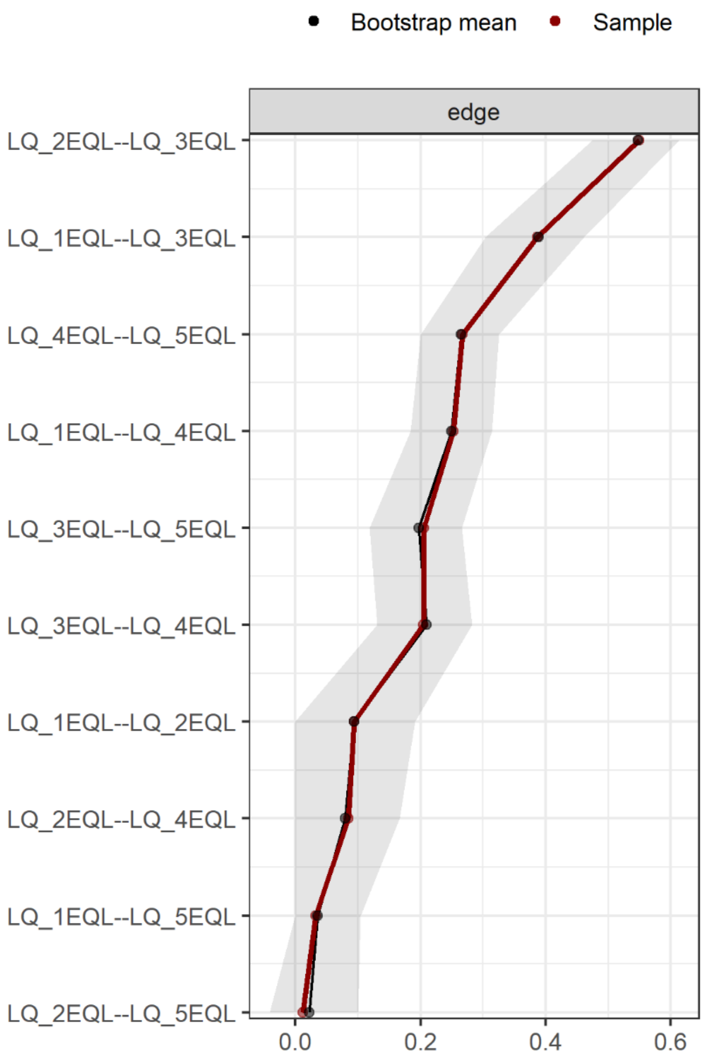

Figure S2

Case-dropping bootstrap graph for centralities, strength, closeness, and betweenness for Network I

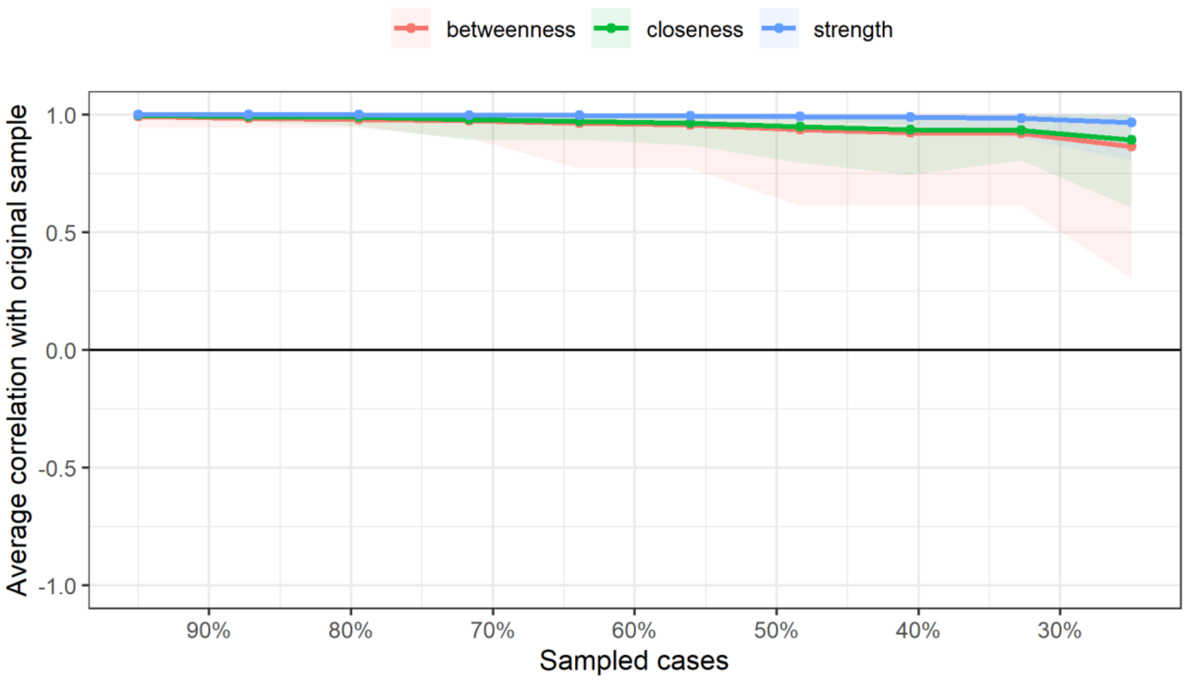

Figure S3

Bootstrapped 95% confidence intervals for all edges in Network II

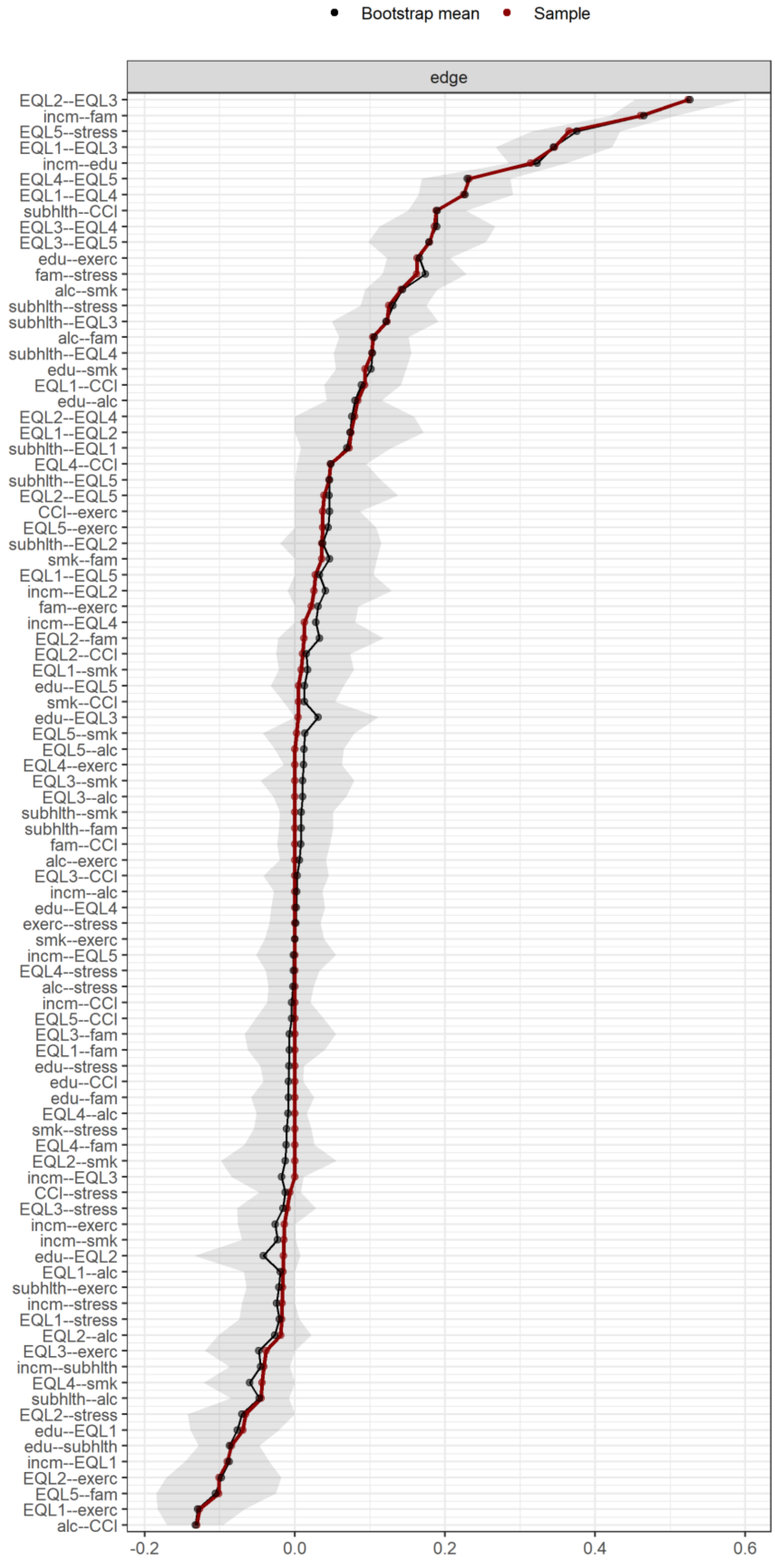

Figure S4

Bridge closeness, bridge betweenness, and bridge two-step expected influence of EQ-5D dimensions and life factors. *X*-axis indicates the original scores of each centrality index. Variables are ordered from largest to smallest, from top to bottom as indicated in the *y*-axis.

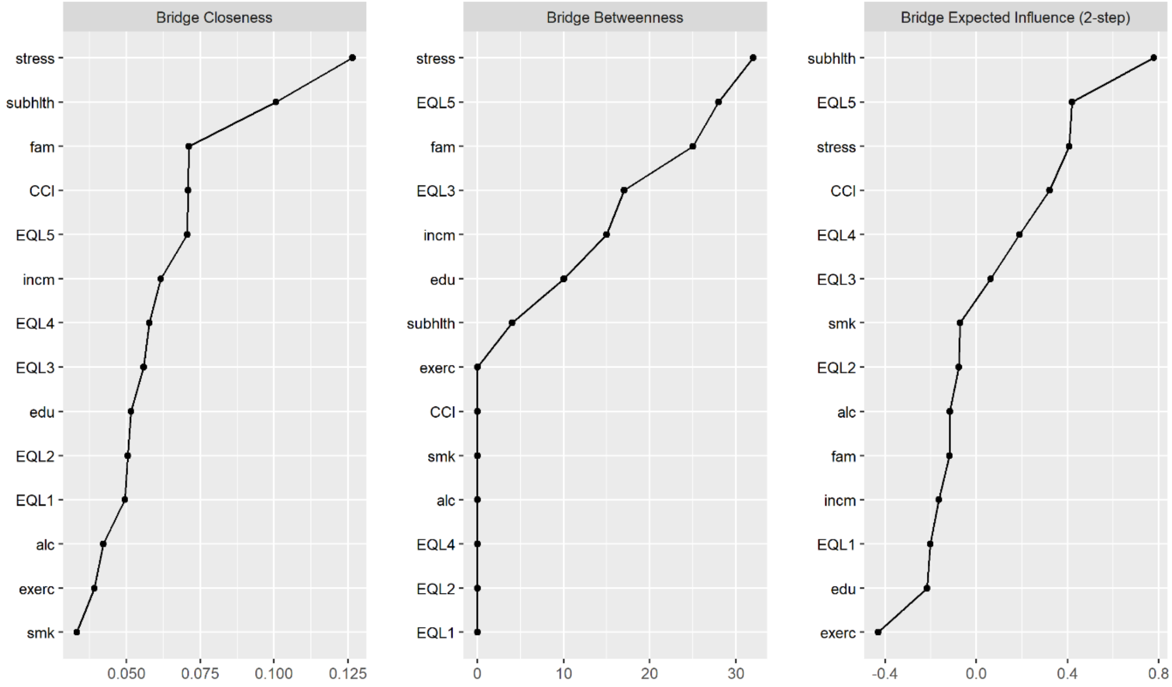

Figure S5

Case-dropping bootstrap graph for bridge betweenness, bridge closeness, and bridge expected influence for Network II

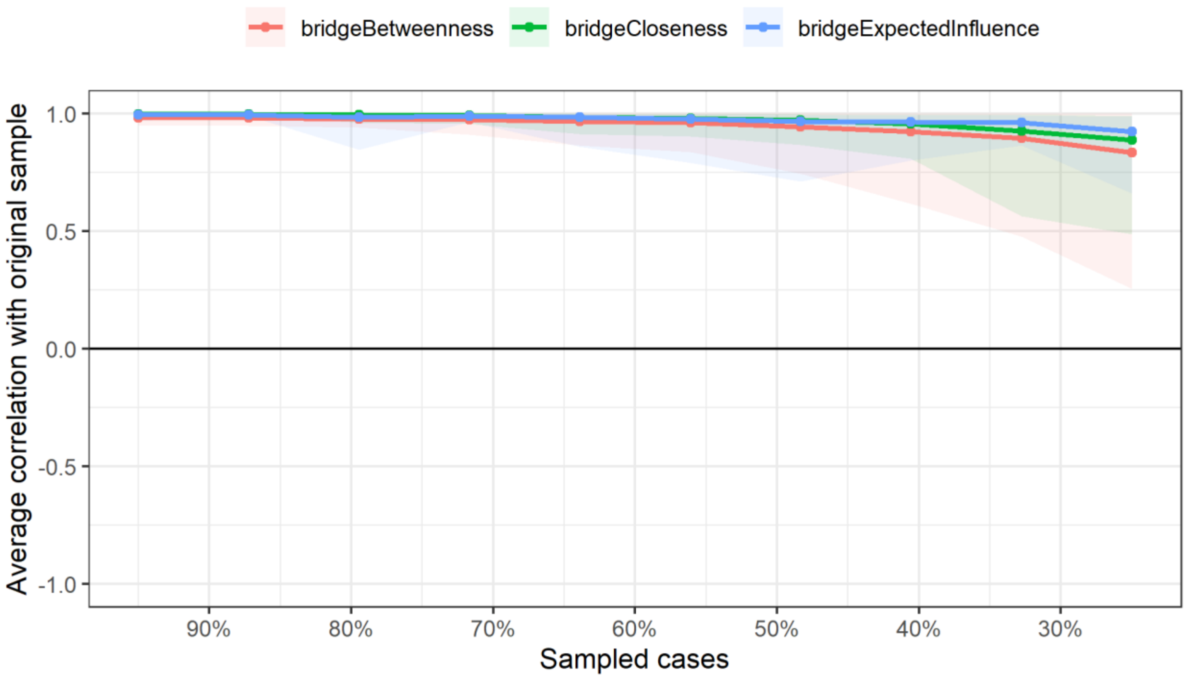

Figure S6

Network III showing EQ-5D index and life and psychosocial factors. Thicker lines indicate stronger edge-weights. Red indicates negative edge-weights and green indicates positive edge-weights.

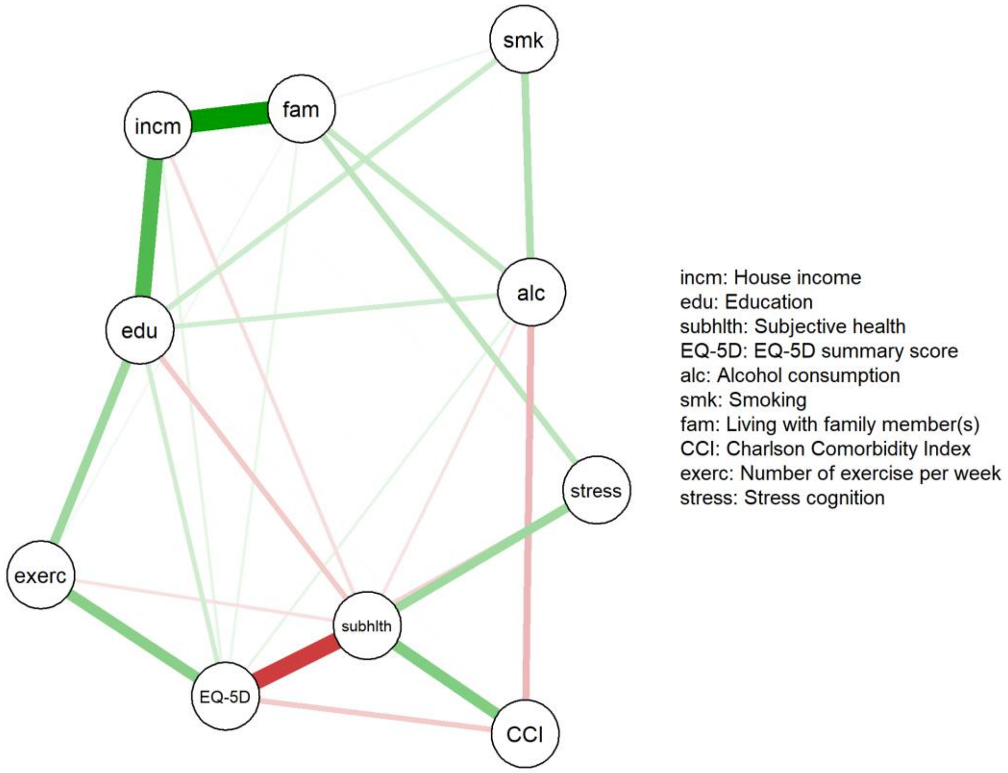

Figure S7

Bootstrapped 95% confidence intervals for all edges in Network III

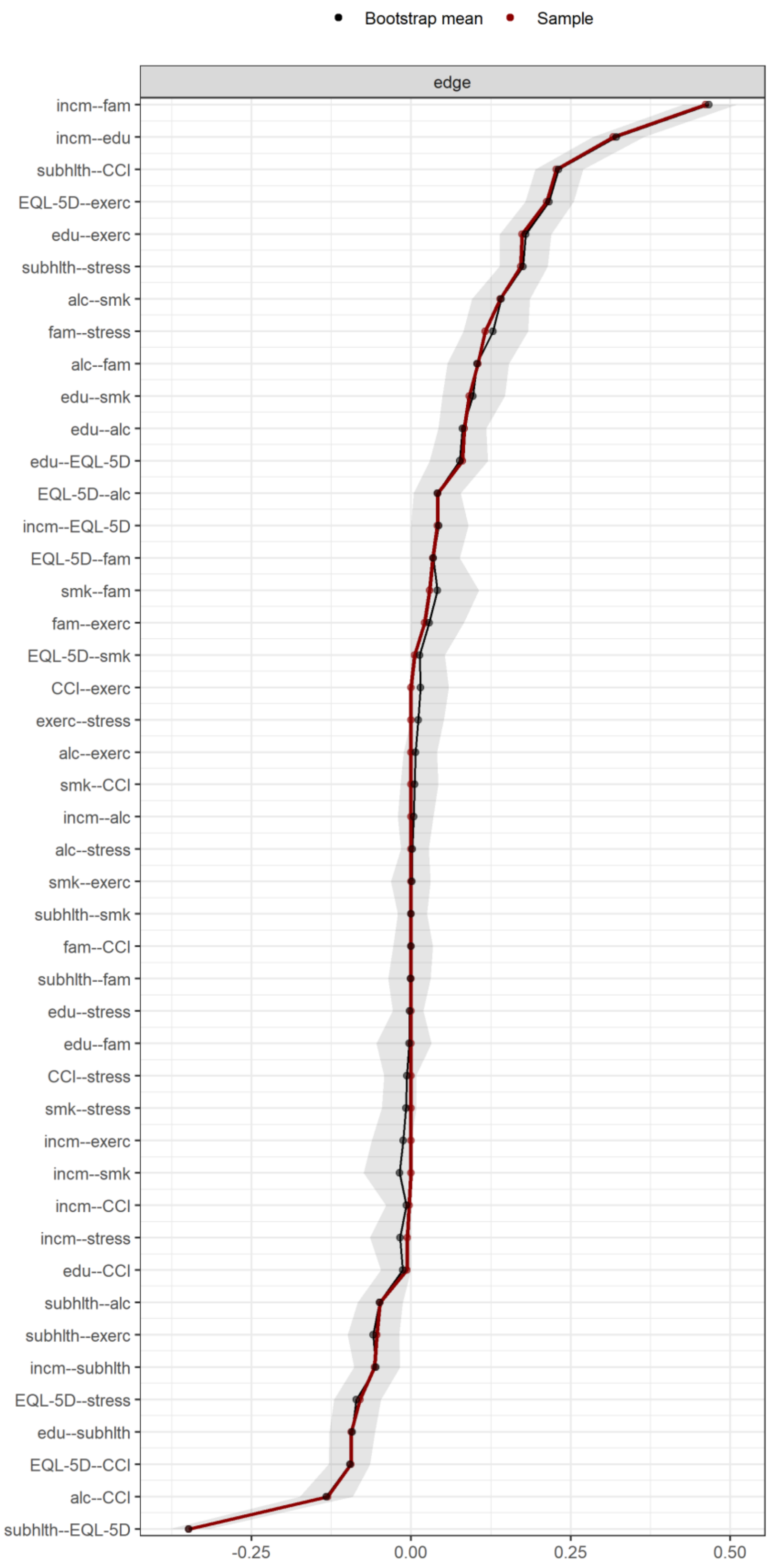

Supplementary Text S1

Network Analysis

As our data is cross-sectional, we estimated an undirected, weighted network using Gaussian graphical model (GGM), where edges represent partial correlation coefficients (Burger et al., 2022; Epskamp et al., 2018). GGM requires the data to have a multivariate normal property. However, in the case of ordinal data, polychoric correlations can be used instead (Epskamp, 2016). The polychoric correlation matrix undergoes a regularization process to create a parsimonious and sparse model by giving penalty for model complexity (Epskamp & Fried, 2018). For GGM with ordinal data, a regularized estimation was conducted using the graphical least absolute shrinkage and selection operator (glasso) with the Extended Bayesian Information Criterion (EBIC) (Epskamp & Fried, 2018; Friedman et al., 2008). This regularization process is included in the *qgraph* package, called the *EBICglasso* function (Epskamp et al., 2012). As a result, spurious edges represented by weak partial correlation coefficients are removed from the network, thus improving its parsimoniousness and sparsity.

For centrality analyses, strength, closeness, and betweenness centrality indices were computed using the centrality function included in the *qgraph* package (Epskamp et al., 2012). Strength centrality is a measure of how strong a node is related to each adjacent nodes by considering the absolute values of edge weights. Closeness refers to the inversed sum length of the shortest path of a node to all other nodes in the network. Finally, betweenness is the number of times a node of interest is passed through in the shortest route between every possible pair of nodes in the network (Bringmann et al., 2019; Opsahl et al., 2010).

Bridge Centrality Analysis

Bridge centralities, including bridge closeness and bridge betweenness were identified using the bridge function in *networktools* package (Jones & Jones, 2017). To account for the presence of both positive and negative edges in the network, we computed a two-step bridge expected influence. Expected influence is a similar measure to strength, but with consideration of negative relationships. A two-step expected influence takes into account not only the edge between focal node and its connected node, but also subsequently connected nodes (Robinaugh et al., 2016). Bridge centrality indices are defined similarly to extant centrality indices, but in the context of node communities.

Accuracy and Stability

For all parameters estimated, we performed bootstrapping to evaluate their accuracy and stability using the *bootnet* package (Epskamp et al., 2018). First, the accuracy of edge-weights of the network was evaluated by estimating the 95% confidence interval (CI) on each edge using non-parametric bootstrap method with 1000 bootstraps. Next, centrality indices were also evaluated where applicable. Stability of centrality indices can be evaluated using the correlation stability coefficient (CS-coefficient). CS-coefficient is obtained by employing the case-dropping subset bootstrap, where portions of cases (i.e. observations from the data) are dropped from the data and correlation between the subset centrality indices and original centrality indices are calculated. CS-coefficient is a measure of the maximum proportion of cases that can be dropped such that the correlation is higher

than the chosen value. Recommended cutoff scores for CS-coefficients is 0.5, and it is advised not to interpret centrality indices below 0.25 (Epskamp et al., 2018).

Network Visualization

Network I was visualized using the *qgraph* function, as Figure 2a. All 10 possible edges are described by partial correlation coefficient regularized using the EBICglasso method, with hyperparameter set to the default value, 0.5 (Epskamp et al., 2012). The strongest edge is the edge connecting EQL2 (self-care) and EQL3 (usual activities), with partial correlation coefficient value 0.55. Nodes were placed with respect to the Fruchterman-Reingold algorithm applied using *spring* layout, where strongly related nodes are placed closer together (Fruchterman & Reingold, 1991). The results for bootstrapped CI for each edge is supported in Supplementary File, Fig. S1.

The centrality analysis revealed that overall, largest centralities were observed for EQL3, usual activities. Figure 2b illustrates the raw centrality scores for strength, closeness, and betweenness. CS-coefficients were 0.75, 0.75, and 0.52, respectively. As all CS-coefficients were above 0.5, all indices were treated as accurate and were thus interpreted. The results for case-dropping bootstrap for the centrality indices can be accessed in Supplementary File, Fig. S2.

References

Bringmann, L. F., Elmer, T., Epskamp, S., Krause, R. W., Schoch, D., Wichers, M., ... & Snippe, E. (2019). What do centrality measures measure in psychological networks?. *Journal of abnormal psychology*, *128*(8), 892.

Burger, J., Isvoranu, A. M., Lunansky, G., Haslbeck, J., Epskamp, S., Hoekstra, R. H., ... & Blanken, T. F. (2022). Reporting standards for psychological network analyses in cross-sectional data. *Psychological Methods*.

Epskamp, S. (2016). Regularized Gaussian psychological networks: Brief report on the performance of extended BIC model selection.

Epskamp, S., Borsboom, D., & Fried, E. I. (2018). Estimating psychological networks and their accuracy: A tutorial paper. *Behavior research methods*, *50*(1), 195-212.

Epskamp, S., Cramer, A. O., Waldorp, L. J., Schmittmann, V. D., & Borsboom, D. (2012). qgraph: Network visualizations of relationships in psychometric data. *Journal of statistical software*, *48*, 1-18.

Epskamp, S., & Fried, E. I. (2018). A tutorial on regularized partial correlation networks. *Psychological methods*, *23*(4), 617.

Friedman, J., Hastie, T., & Tibshirani, R. (2008). Sparse inverse covariance estimation with the graphical lasso. *Biostatistics*, *9*(3), 432-441.

Fruchterman, T. M., & Reingold, E. M. (1991). Graph drawing by force-directed placement. *Software: Practice and experience*, *21*(11), 1129-1164.

Jones, P., & Jones, M. P. (2017). Package 'networktools'. *Available from: https://cran. rproject. org/web/packages/networktools/networktools. pdf.*

Opsahl, T., Agneessens, F., & Skvoretz, J. (2010). Node centrality in weighted networks: Generalizing degree and

shortest paths. *Social networks*, *32*(3), 245-251.

Robinaugh, D. J., Millner, A. J., & McNally, R. J. (2016). Identifying highly influential nodes in the complicated grief network. *Journal of abnormal psychology*, *125*(6), 747.
